# Spinal Cord Infarction Presenting as Right-Sided Upper Back Pain: A Case Report

**DOI:** 10.7759/cureus.30104

**Published:** 2022-10-09

**Authors:** Filipa Madalena F Gonçalves, Ana Luísa Campos, Magda Costa, Isabel Trindade, Jorge Cotter

**Affiliations:** 1 Internal Medicine, Hospital Senhora Oliveira, Guimarães, PRT

**Keywords:** spinal cord infarction, chest pain, emergency department, medullary stroke, ischemic cerebrovascular disease, medullary infarct

## Abstract

Spinal cord infarction is a very rare event with a wide variety of symptoms at presentation. We describe the case of a 39-year-old man who presented to the emergency department with atypical chest pain. The initial investigations were non-diagnostic, and the patient was admitted for surveillance. On the second day of admission, he developed neurologic deficits; a second computed tomography showed a medullary infarction at levels C5-T2. Dual antiplatelet therapy was initiated. An extensive study on the underlying etiology was performed. It was considered to be an idiopathic event. The patient was discharged to a rehabilitation center for bladder training and motor training due to quadriplegia level D on Asia Impairment Scale with a C6 neurological level with left predominance and a hand grip deficit that disabled him to grab objects. This case report describes a rare event with a biphasic ictus at presentation. It highlights the difficulty in managing this pathology because of limited clinical data.

## Introduction

Spinal cord infarction may have a wide variety of symptoms at presentation depending on the affected vascular territory. According to the literature, it is a rare condition, with an estimated incidence of 0.003% of all events, and 0.3-2% of all stroke events [1,2].

In most cases, the etiology is not identified. Causes can be categorized into iatrogenic and non-iatrogenic; the former may be due to aortic surgery or injuries caused by other surgical procedures, while non-iatrogenic causes include trauma, arteriosclerosis, arteriovenous malformations, thrombotic/fibrocartilaginous emboli, polycythemia vera, vertebral hyperextension, myelitis, infections, and/or neoplasms [1,3].

This case report aims to describe a rare case of acute spinal cord ischemia syndrome with an atypical presentation and its management, particularly treatment and etiologic investigation.

## Case presentation

A 39-year-old caucasian man with a history of cigarette smoking, occasional use of cannabinoids, and hypertension presented to the Emergency Department (ED) with spontaneous constrictive pain in the right posterior thoracic region, with acute onset one hour before admission, radiating to the interscapular region and upper limbs up to the elbows, with an 8/10 pain severity, without any history of previous trauma.

**Figure 1:**
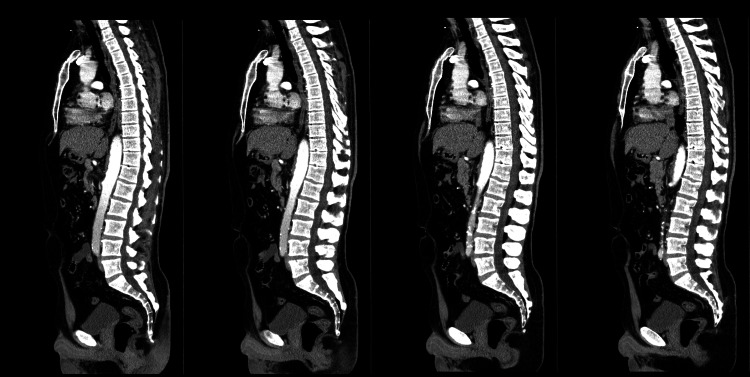
Computed tomography scan at admission in four different sagittal plans.

On admission to the ED, the patient had a blood pressure of 192/113 mmHg, a pulse rate of 130 beats per minute, a regular rhythm, a temperature of 36.8°C, and a respiratory rate of 14 breaths per minute. The physical examination was normal, with cardiac auscultation without murmur, pulmonary auscultation was symmetrical without adventitious murmurs, and no peripheral edema. The neurological examination showed no focal deficits. The electrocardiogram (ECG) showed sinus rhythm, with ST depression in leads V4-V6. A complete blood count and basal metabolic panel were normal, with no elevation of inflammatory parameters (C-reactive protein of 3.2 mg/L), and no increase in cardiac biomarkers (troponin I, creatinine phosphokinase, and myoglobin) at hours zero and six. Additionally, he had a B-type natriuretic peptide level of 43 pg/mL. A transthoracic echocardiogram was performed with documentation of moderate, concentric left ventricular hypertrophy, with no other abnormalities. To exclude an aortic dissection, a computed tomography angiography (angio-CT scan) of the thorax, abdomen, and pelvis (Figure 1) was performed, showing no abnormality. The patient was admitted for clinical surveillance due to persistent symptoms.

**Figure 2:**
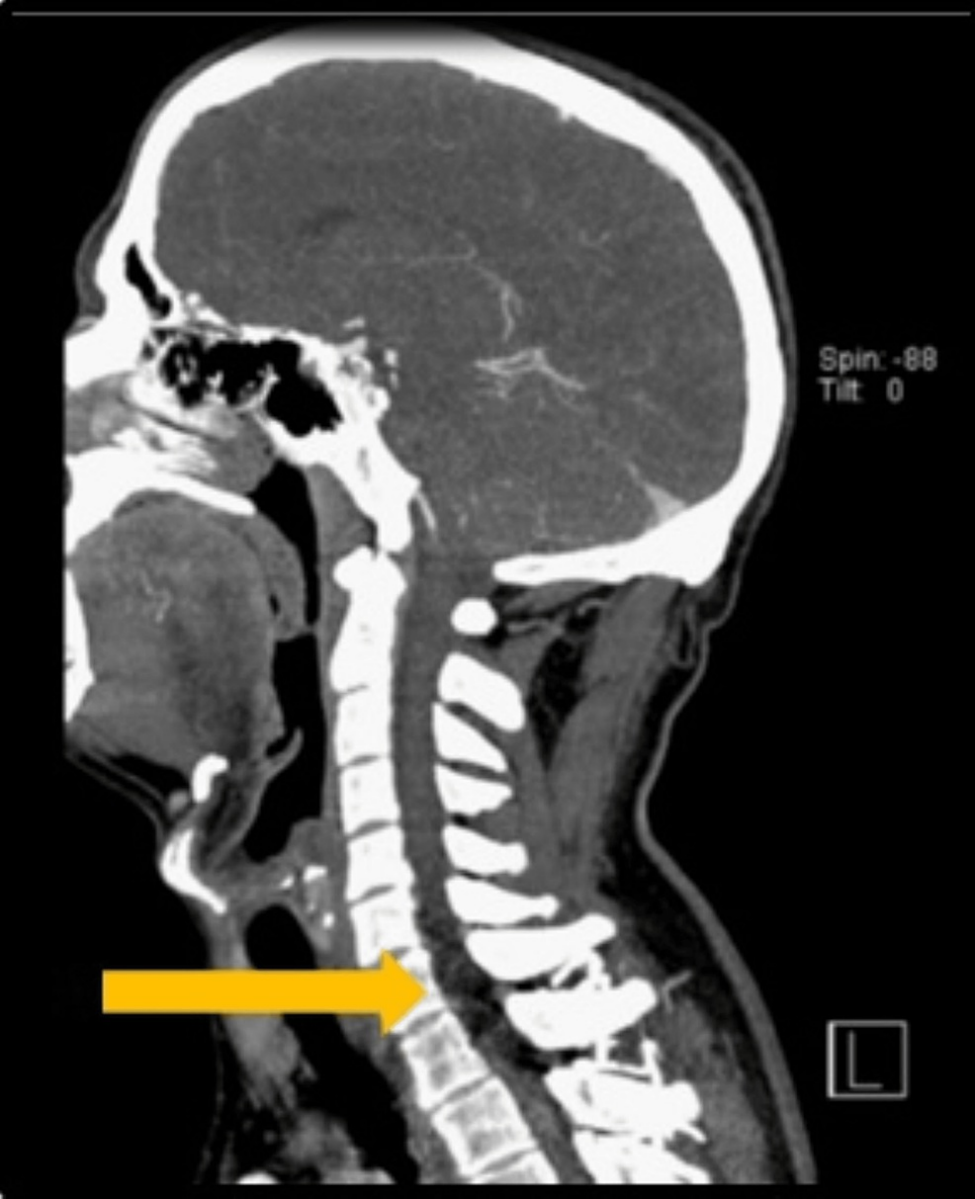
Computed tomography angiography scan showing the lesional area on sagittal view. An arrow pointing to the lesional area extending from C5 to T2.

On the second day of hospitalization, the patient started to have neurological deficits and presented with decreased muscle strength of the left lower limb and both hands with altered sensitivity in the region corresponding to T9-T12 dermatomes, which was associated with acute urinary retention. The neurologic examination revealed myotic pupils, little photoreactive, difficulty in hand grip bilaterally, grade 3/5 muscle strength in the left lower limb, decreased abdominal sensitivity below T5, and bilateral hyporeflexia. Due to the use of morphine for pain control, naloxone was administered without reversal of symptoms and physical examination findings. An urgent angio-CT scan (Figure 2) was performed which showed a lesional area extending from C5 to D2 mostly affecting left anterior and lateral spaces, suggestive of a subacute ischemic spinal cord infarction. Due to subacute evolution and the National Institutes of Health Stroke Scale (NIHSS) score of 3, the patient was not submitted to thrombolysis or thrombectomy, respectively. Dual antiplatelet therapy (DAPT) and a high-potency statin were initiated. Electromyography performed after five days of the initial event revealed increased latency values of F waves of both upper and lower limbs, findings likely related to the involvement of the proximal motor portion of the spinal reflex arc. Moreover, there were signs of non-recent mild neurogenic dysfunction at the level of C7 bilaterally, without activation of voluntary motor units in C8, also bilaterally. In addition, hyporeflexia in the upper limbs distal to C7, as well as areflexia in the lower limbs and sensory level at T3, was noted. In summary, these findings indicated medullary dysfunction.

**Figure 3:**
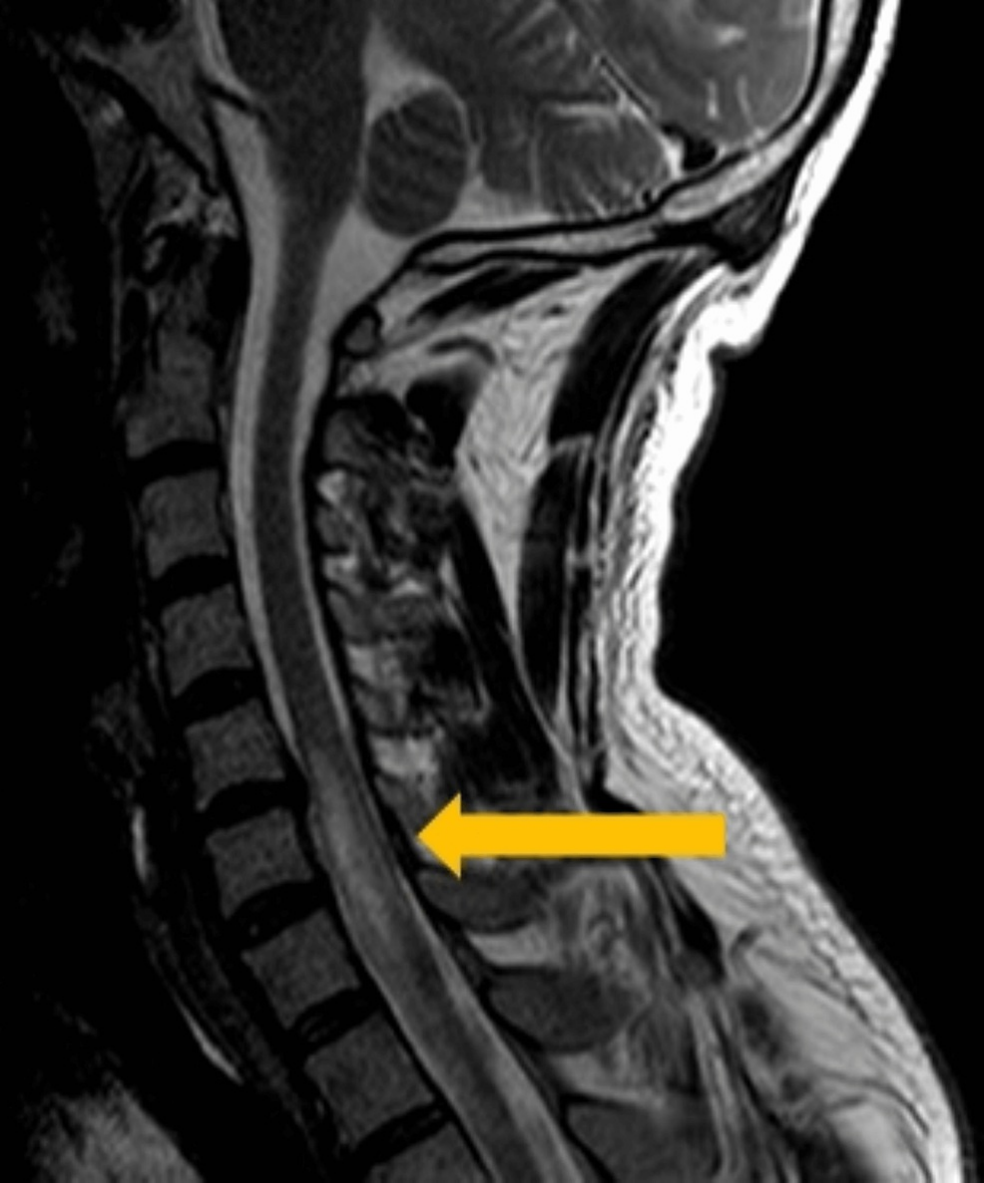
Magnetic resonance imaging showing a spinal cord infarction area on sagittal view. An arrow pointing to the spinal cord infarction area extending from C5 to T2.

A subsequent etiological investigation was carried out. Specific blood tests ruled out autoimmune diseases or hypercoagulable states as antinuclear and antineutrophil cytoplasmic antibodies, lupus anticoagulant, and antiphospholipid antibodies were negative; C protein, S protein, antithrombin III, fibrinogen, and VIII factors were normal; and prothrombin gene and factor V Leiden mutations were noted. Furthermore, lipid profile was normal (total cholesterol, 171 mg/dL, high-density lipoprotein (HDL) cholesterol, 28 mg/dL, low-density lipoprotein (LDL) cholesterol, 95 mg/dL, triglycerides, 237 mg/dL), and glycated hemoglobin was 5.5%. Although the patient was described previously as hypertensive, during hospitalization, he maintained a controlled blood pressure profile without the introduction of antihypertensive drugs. Cranioencephalic and neuraxial magnetic resonance imaging (MRI) (Figure 3) confirmed the diagnosis and showed no vascular malformations. Carotid and vertebral echo Doppler were performed, showing normal permeability of the lumen, with no atherosclerotic disease. A transesophageal echocardiogram ruled out a patent foramen ovale, septum defects, or vegetations. During admission, the patient was electrocardiographically monitored for 72 hours with no evidence of arrhythmias.

During hospitalization, he had nosocomial pneumonia as a complication, with no associated respiratory failure, having completed antibiotic therapy with clinical improvement. The patient started rehabilitation treatment for bladder training due to recurrent urinary retention after an attempt of removing vesical catheterization, as well as motor training due to quadriplegia level D on Asia Impairment Scale with C6 neurological level with left predominance and a hand grip deficit that was disabling to grab objects, with no altered proprioceptive sensitivity. The patient was discharged after 15 days of hospitalization and referred to a motor and functional rehabilitation center.

## Discussion

Spinal cord infarction is often missed in the ED due to its low incidence [1]. In almost 70% of non-specific chest pain may be the initial presentation, and, in these cases, it is localized at the level of the ischemic lesion [4]. Bladder dysfunction is present in more than half of cerebral strokes but uncommon in medullary infarction; however, when present, it is usually associated with urinary retention [5].

Predisposing conditions include aortic aneurysms, venous thromboembolism, coagulopathies, and aortic surgical procedures [4]. The cardiovascular risk factor that appears to be associated with this condition is atherosclerosis, although due to its rare incidence, it remains unclear [2,6]. It has been described as a case of embolization from patent foramen ovale [7]. Classic risk factors such as hypertension and diabetes mellitus are associated with more severe medullary infarction [2].

The evolution of symptoms often occurs within hours. In almost 50% of patients, a biphasic ictus is seen; usually, a radiating pain between shoulders preceding acute or transient sensory spinal cord deficit symptoms. The peak of symptoms can have a duration that can last 35-45 minutes to 24 hours [2]. Initial imaging (CT scan or MRI) may be normal for several days [3,8].

There are no guidelines for the treatment of medullary strokes, and they should be treated as cerebral strokes [2]. Antiplatelets, anticoagulants, when clinically justified, and control of cardiovascular risk factors are the main strategies. Other approaches may be necessary and should be considered individually, such as steroids to reduce medullary edema and/or cerebrospinal fluid drainage. There are no data based on clinical trials that support the use of fibrinolysis [3,8].

The severity of symptoms at presentation is the prognostic factor with a more long-term predictive value [1]. Men and young adults tend to present with more severe symptoms; however, they tend to improve more rapidly. Rehabilitation programs tend to have a favorable outcome. Autonomic dysfunction and chronic pain are reported in most patients on follow-up [2,4].

## Conclusions

The diagnosis of spinal cord infarction is very challenging due to its low incidence and the variety of symptoms that can be present at admission. Biphasic ictus and normal initial imaging, as seen in this case, might be another factor contributing to the delay of diagnosis, and, therefore, management and treatment. Idiopathic events are common despite a long and exhaustive study.

Antiplatelets/anticoagulants, control of cardiovascular risk factors, and rehabilitation are the main strategies for management and follow-up in these patients. Although fearful, this entity usually has a better long-term outcome.
